# Supplementary figures and images for: Decomposing spontaneous sign language into elementary movements: A principal component analysis-based approach
Source: PLoS One. 2021 Oct 29;16(10):e0259464. doi: 10.1371/journal.pone.0259464 (PMC8555838; doi:10.1371/journal.pone.0259464)

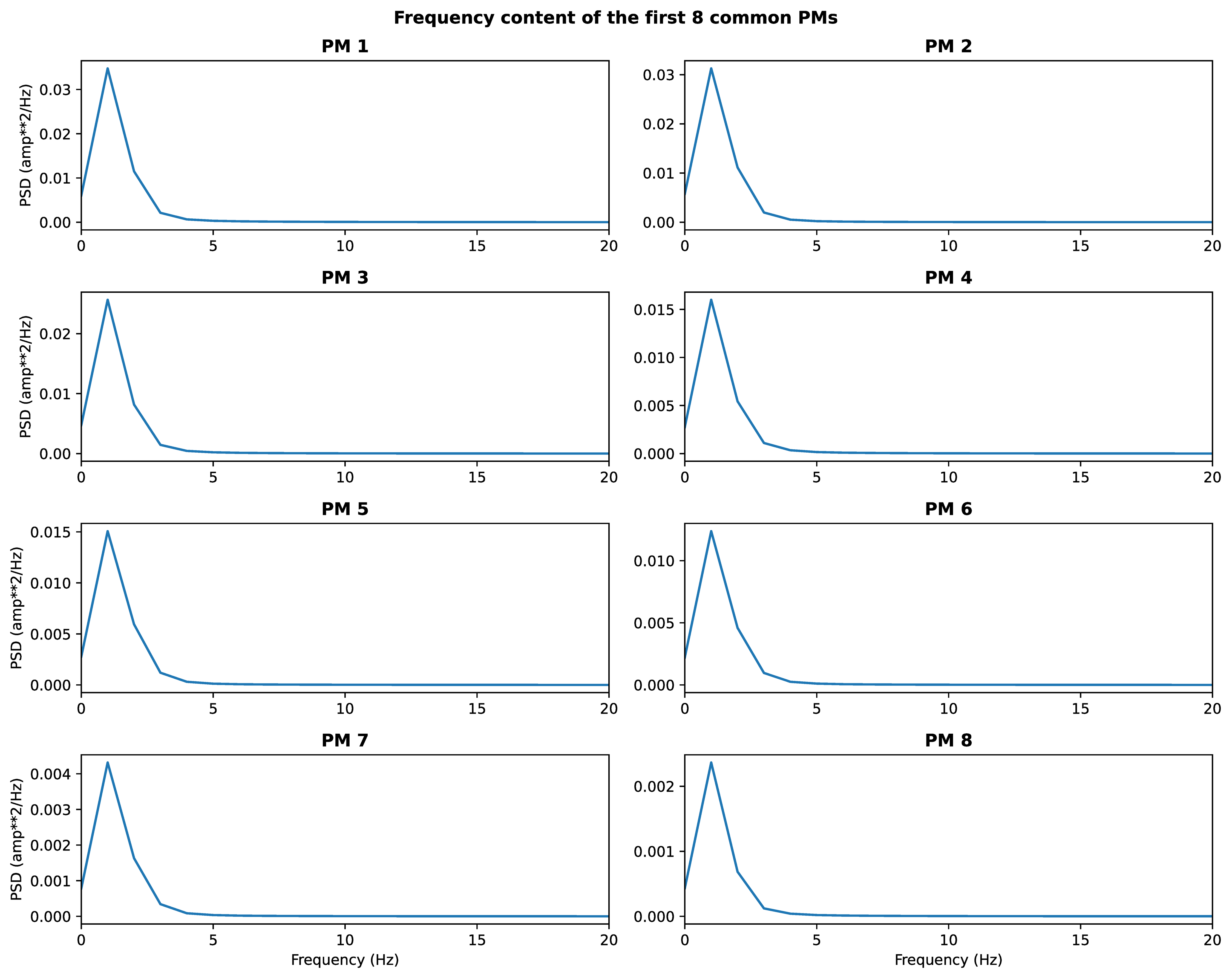

Supplement: S1 Fig — Power Spectral Density was estimated using the Welch method. (TIF) [file pone.0259464.s001.tif]
