# Supplementary material for: Decomposing spontaneous sign language into elementary movements: A principal component analysis-based approach
Source: PLoS One. 2021 Oct 29;16(10):e0259464. doi: 10.1371/journal.pone.0259464 (PMC8555838; doi:10.1371/journal.pone.0259464)
Supplement: Attachment — Submitted filename: Response to Reviewers.pdf [file pone.0259464.s003.pdf]

We would like to thank the reviewers for their thoughtful and constructive comments. The points raised in the reviews were all of high relevance and we have made significant modifications and additional work in order to address them. In summary:

- A significant effort has been made to motivate our aims as compared to prior work related to SL and to refine the discussion with prior work successfully applying PCA to human motion in general. The key original contributions of the paper were made clearer, including in the last sentences of the abstract.
- Statistical analyses were run on individual and common PMs in order to quantitatively assess the consistency of PMs across signers (i.e., leave-one-out cross-validation, cosine similarity between PMs and cross-projection similarity between PM subspaces specific to each signer).
- 2 additional markers have been included in the analyses in order to better capture torso movements (this may have had some effect on PM8, but did not impact the rest of the findings).
- Visualization of inter-individual differences in common PMs was removed, as it does not add relevant insights into the key problem of the paper.

+ the custom code used in the study has been shared, in order to make our implementations available to the reader (ln. 128-130).

We provide further detailed answers to the reviewers' comments below:

Reviewer #1:

Dear Authors,Thank you for submitting this manuscript and for your contribution to the movement science field overall. I have decided that 'Major Revisions' are required for the following reasons in no particular order:

The main focus and finding of this study, that sign language movement is constrained to a low dimensional space isnt a novel finding (https://doi.org/10.1038/s41467-020-17404-0) but what is interesting is that it replicates this finding with a more liberal dataset where participants were more self-directed. As you will see in this paper also, PCA was applied successfully, making the main findings here rather redundant. An effort should be made to build on previous work or make a direct connection to related work and how it has been supported or not.

Thank you for this comment of high relevance, and for the missing reference. The paper (+ other older studies on hand gestures in ASL letters) has been included and the outcomes further discussed (see ln. 59-95 in the Introduction, ln. 280-369 in the Discussion). As you have pointed out, our study differs from these SL-related studies in one crucial respect: we analyzed unconstrained continuous SL by contrast with ASL letters, whose movements are highly constrained and hardly reflect SL in real-life conditions. First, spontaneous SL gives rise to a far wider variety of gestures (involving various linguistic forms). Furthermore, it is known that SL has specific properties when produced in context (notably due to co-articulation) rather than isolated (refs 34-36). We highlighted this crucial need to study continuous SL in order to gain further fundamental insights and in order to improve real-life SL automatic tools. Furthermore, we outlined how our analyses interestingly complement those previous studies in another respect: SL is not limited to the dominant hand gestures

(it also involves the other hand, torso, head, arms…). It is therefore interesting to focus on both approaches (global upper-body motion in our study, finger gestures in prior work).

The variance accounted for as a metric of complexity is not very comparable across datasets as it is sensitive to various non-movement related differences including noise, number of markers included etc. Therefore, premising the study on this finding is valid when making comparisons within the same dataset but is not advised across datasets. There is no justification explicitly provided for the number of synergies extracted. Your results are as one would expect, that sign language movements are one of the more complex of movements but using skiing as a comparison here isnt very informative. There are a number of hand gesture related studies in the literature that may make for more insightful comparisons for the underlying motor control mechanisms. Linking the findings back to how they may help with the greater goal you have mentioned (technology development) throughout the paper will drive home the point of the study also.

We agree that comparing the exact number of PMs retained across datasets was confusing. We considerably reduced the level of detail of such comparisons, especially since it does not add substantial information toward the key message of the paper: upper-body motion of spontaneous SL is constrained to a low-dimensional subspace (regardless of the exact number of PMs retained). We think it's still worth mentioning other findings made on full-body motion, in addition to hand gestures as you suggested (in particular ones related to SL) (ln. 280-301), as no prior work has been done on upper-body movements of SL. As mentioned before, we focused on upper-body motion as SL is far from being limited to gestures of the dominant hand. It may thus be informative to discuss prior work that included full-body mocap but not in SL, as well as prior work that included finger mocap in SL. Still, comparing the exact numbers of PMs may be counterproductive and we agree that using it as a metric of complexity is not that relevant when done across datasets. A more relevant observation on PMs and complexity was done within our dataset: that the similar number of PMs across signers could reflect their similar level of expertise (ln. 331).

Although higher order PMk can contain subtle movement characteristics as you have shown, these are also vulnerable to noise and so it may be worth including in your study an analysis of the signal strength in these synergies.

We totally agree with this observation. In order to be consistent with prior analyses of the signal strength in PMs (e.g., 10.3389/fnagi.2018.00022), we extracted the PMs from the raw mocap data and then assessed their frequency content. Finally, the PM scores were low-pass filtered accordingly (< 6Hz), then the effect of noise was deemed to have been sufficiently reduced (ln. 167-174). Please note that there may not be a huge difference in this respect between the PMs of this new version and of our first analysis, as in the first we had low-pass filtered the raw mocap data with a cut-off frequency of 12 Hz–which is already far fewer than the original maximum frequency 125 Hz– and then extracted the PMs. Still, this newer version allowed refining the cut-off frequency to 6 Hz and we also think that following a consistent

procedure (i.e., scrutinizing the spectral content of the PMs) as compared to prior studies makes our purpose clearer.

If you would like to say that these synergies were consistent across participants, and wouldnt drastically change if a participant was added/left out, this approach is advisable, and would make the results generalizable. One method for this has been conducted in the research you cite, a leave one out cross validation (10.3389/fnagi.2018.00022).

Thank you for suggesting this validation step, which we think is even more than a validation step in the present purpose, as it is clearly a first way to assess the extent to which PMs extracted in spontaneous discourses could be shared across signers (one of the aims of the paper). A leave-one-out cross-validation was conducted (ln. 175-187) and revealed that the first eight PMs (explaining 94.6% of the variance) were robust (on average across signer changes, the PC-vectors changed their orientation by less than 15°). PM5 and PM6 were slightly less robust in the specific case where Signer 5 was left out, which motivated further analyses quantifying the consistency of the PMs across signers (see below).

Although you aim for a qualitative analysis, this may be difficult when properly addressing your aims and describing the results of a computational method while adding to the existing knowledge base. The capacity for the PCA protocol to capture individual differences and common synergies is well known, and the amount of words spent on this is not justified based on the existing evidence base. The similarities and differences between individuals is indicated using discrete examples but not quantified, and goes against a motivation of the study which was to holistically quantify movement parameters. Perhaps illustrating some metrics for how this approach has holistically captured the movements rather than discrete examples would be more informative. Again, linking these findings to those in the literature related to sign language is advisable. Although you aim for a qualitative analysis, perhaps an exploratory approach that involves a mixture of qualitative and quantitative findings would be more suitable for addressing the aims of the study. The following paper may be of use for such an approach: doi: 10.3389/fspor.2020.596063. Much of this analysis could have been conducted using the PManalyzer software (10.3389/fninf.2019.00024), if this was indeed used, please cite.

Thank you indeed for the suggested papers. In addition to the first leave-one-out cross validation (which provided first insights into the consistency of PMs across signers), we conducted two statistical analyses in order to provide quantitative evidence that PMs (or at least PM subspaces) were similar across signers: cosine similarity between individual and common PMs, and cross-projection similarity between individual PM subspaces (ln. 187-214 in Methods, ln. 242-278 in Results). The first metric allowed confirming the observations made on discrete examples in the first manuscript (i.e., a consequent number of individual PMs are highly similar to the common PMs) quantitatively. The second one brought interesting further insights into the similarity of the PM subspaces: individual PMs could be ranked in a different order or be expressed as combination of common PMs and thus potentially report

low cosine similarity values. However, when computing cross-projection similarity, it revealed that the PM subspaces (spanned by the first 8 PMs) specific to each signer accounted for most of the variance in the movements of the other signers, revealing similar low-dimensional spaces across signers used to produce the complex SL motion.

Please proof read the manuscript for spelling and grammar and reference using a single format throughout the paper.

This revised manuscript has been proof-read.

Overall the study has the potential for publication if the aims of the paper are adequately addressed, the methodology and results are properly justified and generalizable, comparisons are made within context and a consistent message is provided throughout the paper of novel results.I look forward to reading your revised version.

Reviewer #2:
Summary:The manuscript „Decomposing spontaneous sign language into elementary movements: a principal component analysis-based approach" describes how the French sign language of six participants can be decomposed into principal movements (PMs) using motion capturing data and PCA as decomposing method. One finding was that the first eight PMs, which were calculated considering the data of all six participants, reflected 94.9% of the overall variance. Moreover, the first eight PMs calculated individually for every participant reflected 95.9% (SD = 0.6%). Since the movements of the different participants were not synchronized, the authors interpreted this similarity by stating that PCA is a valid method to decompose sign language. Moreover and following the argument of the authors, sign language appears to follow dominant movement patterns which are similar upon different signers. The manuscript is well written besides some small misspellings. However, there are some major and minor concerns which I would like to address below.

Major Concerns:

Abstract: The last sentence says that the results suggest "that SL may have a common structure…". Isn't that obvious since it is supposed to be understood by different people who potentially never met each other, but who use the same technique to communicate? I miss a statement about the importance and relevance of the findings. What does this finding indicate or which implications could be formulated based on the results? Was the only purpose of the study to validate if PCA is a method to decompose sign language? If yes, please clarify why this is of importance.

Thank you for raising this lack of clarity, in particular in the last sentences of the abstract, which have now been refined. More generally, significant efforts have been made throughout the paper to clarify why this study is of importance. We also think that the specific formulation "SL may have a common structure" was confusing. To

clarify further, the fact that upper-body movements of different signers in spontaneous, unconstrained, SL are constrained to a low-dimensional space was not obvious (see notably ln. 302-320), although it was expected that such movements were optimized by signers and that some dimension reduction could thus be found. In prior SL-related studies on hand gestures of ASL letters (i.e., highly constrained gestures) (refs 30-33), one could effectively expect to find common synergies as all signers performed gestures that had to conform to the well-defined forms of a common alphabet (as stated by the authors themselves, in ref 30). By contrast, the signers of our study could express themselves freely and thus use a virtually infinite range of linguistic forms (thus different gestures), which makes our final results (i.e., a few PMs shared across signers can account for the variance in the motion) not trivial. Studying spontaneous SL is crucial in SL research as signs made in isolation (e.g., refs 30-33) hardly reflect the complexity of SL movements in real-life conditions (see ln. 291-301). Furthermore, SL is not limited to the dominant hand gestures studied in the prior SL-related work (refs 30-33): it also involves the other hand, torso, head, arms... It is therefore interesting to focus on the movements of further upper-body parts. The purpose of this study was therefore to test if spontaneous upper-body movements of SL can be described within a low-dimensional subspace despite the high number of potential linguistic gestures, and to assess the extent to which PMs were consistent across signers and discourses. We think that this study will have significant contributions to SL research (1) for fundamental purposes about the control of SL in real-life conditions; (2) in order to provide efficient real-life automatic SL processing tools that rely on heavy 3D mocap datasets (see notably ln. 371-394).

Methods: Did the participants write an informed consent and/or was the procedure approved by an Ethics committee?

Yes, all participants gave informed written consent (translated into LSF for better accessibility, as they were all deaf) before the experiment, in accordance with the ethical standards of the Declaration of Helsinki. We added this information (ln. 111-112).

Line 113: The authors state that normalization took place by subtracting the participants average posture from the posture vectors, which is more a centering of the data and does not ensure completely that each participant contributes the same variance to the newly created matrix (see reference 21). I would suggest to also normalize the vectors afterwards by dividing their mean Euclidean distance, also described in reference 20 and 21. Especially for the PCA calculated for all six participants, this would be crucial to only “extract common PMs” as stated in line 141.

Yes, thank you for this highly relevant comment. We conducted the two-step normalization (ln. 128-155) following the suggested prior paper (ref 23). We agree that such normalizations (e.g., reducing anthropometric differences) are crucial when extracting PMs across different individuals. Please note that in the first version of our analysis, we were not just subtracting the participants average posture, but we were then additionally replacing it by the “mean average posture” computed across all signers (similarly to the shape normalization of [Troje et al., 2005, Perception &

Psychophysics]), which allows reducing anthropometric differences more than a simple centering of the data. This could explain that our revised results seem similar to the ones of our first version. Still, it's probable that dividing by the mean Euclidean distance allowed further normalization (a comparison between the two normalization methods is out of the scope of this paper), and, more importantly, we think that it's clearer to follow the exact same procedures as in prior papers that are the references in the study of PMs and motor control strategies (e.g., ref 23). We therefore decided to center and then normalize the data to the mean Euclidean distance.

Were the markers weighted based on their relative body mass, e.g. as defined by de Leva?

No, the markers were not weighted based on the relative body mass of the corresponding segments. Although including the segments body mass in the normalization could be interesting for SL research, we here aimed for a kinematic analysis (similarly to prior work, such as refs 19, 21, 23, 46), by contrast with other studies focusing on forces or accelerations (like refs 24, 26 or 51), where the normalization based on the relative mass of the segments may be necessary. But in this respect, we think that the present outcomes call for further analyses of SL movements investigating postural accelerations and, in that case, markers would need to undergo this normalization.

Line 151: No statistical analysis was conducted which in my opinion makes it difficult to objectively evaluate the findings of the study. Yes, taking a look at the visualizations or the figures, the PMs appear to be quite similar. However, to state that the "PMs describe highly similar motion patterns" (line 283) is a very subjective statement in my opinion and should be tested using statistics. E.g. tests for correlation could be applied on the postures during the PMs as shown in Figure 4.

Yes, thank you for this constructive comment and for the suggested metric (we did not use correlation directly but cosine similarity, which captures very similar information). First, we performed a leave-one-out cross validation (where one signer was added or left out, ln. 175-187), which revealed that the first eight PMs (explaining 94.6% of the variance) were robust across signers. We then conducted two statistical analyses in order to provide further quantitative evidence that PMs (or at least PM subspaces) were similar across signers: cosine similarity between individual and common PMs, and cross-projection similarity between individual PM subspaces (ln. 187-214 in Methods, ln. 242-278 in Results). The first metric allowed confirming the observations made on discrete examples in the first manuscript (i.e., a consequent number of individual PMs are highly similar to the common PMs) quantitatively. The second one brought interesting further insights into the similarity of the PM subspaces: individual PMs could be ranked in a different order or be expressed as combination of common PMs and thus potentially report low cosine similarity values. However, when computing cross-projection similarity, it revealed that the PM subspaces (spanned by the first 8 PMs) specific to each signer accounted for most of the variance in the movements of the other signers, revealing similar low-dimensional spaces used across signers to produce the complex SL motion.

Line 283: Following my comment stated before, to me it is not possible to make such a statement based on the results presented in the manuscript as they are just based on descriptive evaluation.

We agree and believe that the combined three statistical analyses included in the revised manuscript make this statement justified (ln. 340).

Minor Concerns:

Line 11: The authors mention the importance of hands, torso and face for SL. I see that it is difficult to evaluate the mimic in the face and that it is not the focus of their study. However, why was the amount of markers reduced from 27 to 19 (line 92)? That takes away the chance to take the torso movement into account.

Yes, your comment raises some lack of clarity in the method description. Across all the six signers, only 23 markers were available (27 was the maximum number of marker available for some signers, but it was unfortunately not the case for all of them). However, we agree with the issue that it may have compromised the inclusion of the torso movements, a problem that we were able to overcome by including the two markers of the torso (included in the total 23 markers, but that we had removed to ease visualization). Thank you for this remark, which we think has allowed highlighting torso movements in the 8th PM. Moreover, of the 23 markers available for all signers, we chose to represent each elbow using one marker instead of two (it did not add substantial information and confused visualizations), which resulted in the final 21 markers. All these comments were clarified in ln. 118-121.

Line 41: Misspelling: “Egeinvoices”

Thank you, this is rectified.

Line 58-61: PCA was also already used to search for and find changes in postural control due to impairments like cerebral palsy (Rethwilm, R., Böhm, H., Dussa, C. U. & Federolf, P. (2019). Excessive lateral trunk lean in patients with cerebral palsy: is it based on a kinematic compensatory mechanism? Frontiers in bioengineering and biotechnology, 7, 345.) or after head shaking (Wachholz, F., Kockum, T., Haid, T. & Federolf, P. (2019). Changed temporal structure of neuromuscular control, rather than changed intersegment coordination, explains altered stabilographic regularity after a moderate perturbation of the postural control system. Entropy, 21(6), 614.). Two articles that might fit in this context as well, as being deaf can be related to a neurological impairment, too.

Thank you for these additional references, which effectively fit in the context of using PCA to study biomechanical strategies through the execution of human movements (ln. 52-54). We think that these specific papers (or papers using PCA to study pathological movements) are interesting with respect to the potentials of PMs to

capture critical information about one's movements. However, being deaf is rarely related to a neurological impairment (e.g., more often related to damage in the ear), or at least it hardly has a direct link with motor neuronal impairments, which makes the study of SL quite distinct from that of pathological movements in this respect. Moreover, SL-specific mechanisms have priorly been reported for deaf but also hearing SL users, by contrast with non-signers (e.g., hearing and deaf signers show higher accuracy at recognizing faces than hearing non-signers [Stoll et al., 2018, Journal of Deaf studies]). This suggests that SL acquisition could influence brain mechanisms, including motor control, beyond differences between deaf and hearing people, which, we agree, is a fascinating question (e.g., how does the linguistic property of SL impact motor control? [refs 43, 57]).

Line 85: I miss some descriptive data about the participants here. I found the average age in reference 26, but could not find any information about the sex, about the inclusion criteria and, if existent, the exclusion criteria.

The missing information was added (ln. 107-111): of the 6 signers, 3 were males and 3 were females. There were only inclusion criteria: the signers had been selected so that they were all deaf, fluent in LSF and working in professions that require them to be comfortable expressing themselves in front of a camera (e.g., teacher, journalist, story-teller, translator).

Moreover, is it valid to talk about "common" PMs, when only six participants were assessed?

That's a relevant remark. Common PMs were termed "common" to distinguish them from individual PMs computed on the separate data of each signer, similarly to Federolf et al. (ref 18) who extracted "common PMs" of skiing across a similarly low number of six athletes. However, we agree with this limitation and thus further outlined that it could be overcome through further investigations (ln. 370-372).

Line 98: Which program was used to calculate the PCA? Was it a custom-made code or an already existing one?

We used custom code for all analyses of the paper, which we made publicly available (ln. 128-130). The precise application of PCA was done using singular value decomposition. All code was written in Python.

Line 121: Misspelling: "Egeinvectors"

Thank you, this is rectified.

Line 178: I mentioned it in the major concerns already, but again: Did the authors perform any statistical test to validate their descriptive interpretation?

We totally agree with the need for further quantitative evidence from statistical analyses, which we added and detailed above.

Line 264: I don’t think that the comparison leading to this result is valid. Skiing is a movement which involves a large amount of joints and therefore results in more degrees of freedom. This ultimately results in a higher number of PMs, which are needed to describe the movement compared to SL – or at least to SL analyzed in the current manuscript – as only the upper body was considered.

We agree that comparing the exact number of PMs retained across datasets was confusing. We considerably reduced the level of detail of such comparisons (ln. 280-301), especially since it does not add substantial information toward the key message of the paper: upper-body motion of spontaneous SL is constrained to a low-dimensional subspace (regardless of the exact number of PMs retained).

General comment: Thank you for the animated visualizations, they help to get a better understanding of the PMs. One suggestion is to implement the name of the PM that is visible and the participant number into the video or at least name the video-file which shows PM1, e.g. ”pm1.mp4”. I assume that “video1” shows PM1 but to avoid misunderstanding I would propose to name the files accordingly to their PM.

Thank you for this suggestion. The videos now have the relevant information in their names (e.g., “video1-commonPM1.mp4”) as well as into the video, which makes their understanding way clearer.
